# Performance and carcass traits of nursery, growing, and finishing pigs fed fourth-generation phytase superdoses in diets severely restricted in calcium and phosphorus

**DOI:** 10.1371/journal.pone.0361635

**Published:** 2026-11-05

**Authors:** Cristina Satie Hideshima Marques, Marco Aurélio Callegari, Cleandro Pazinato Dias, Claudia Cassimira da Silva Martins, Vitor Barbosa Fascina, Alexandre Oba, Rafael Humberto de Carvalho, Caio Abércio da Silva

**Affiliations:** 1 Graduate Program in Animal Science, State University of Londrina, Londrina, Paraná, Brazil; 2 Akei Animal Research, Fartura, São Paulo, Brazil; 3 DSM Nutritional Products Ltda., Jaguaré, São Paulo, Brazil; 4 Novozymes Latin America Ltd., Araucária, Paraná, Brazil; University of Agriculture Faisalabad, PAKISTAN

## Abstract

This study evaluated the effects of increasing inclusion levels of a bacterial phytase in diets with reduced calcium (Ca) and phosphorus (P) on growth performance and carcass traits of pigs across nursery, growing, and finishing phases. A total of 250 pigs (21 days of age; 6.079 ± 0.744 kg) were assigned to a randomized complete block design with five treatments: a positive control (PC), a negative control (NC; −0.18% available P and −0.16% Ca), and NC diets supplemented with 600, 1,200, or 1,800 FYT/kg. During the nursery phase, pigs fed the NC diet showed reduced average daily gain (ADG) and poorer feed conversion ratio (FCR) compared with PC (p < 0.05), whereas phytase supplementation restored performance to levels comparable to PC. Average daily feed intake was not affected (p > 0.05). In the growing and finishing phases, performance variables were affected by treatment (p < 0.05), with NC pigs showing lower ADG and worst FCR, while phytase-supplemented pigs performed similarly to PC. Across periods, quadratic responses to phytase inclusion were observed for FCR (p < 0.001), with estimated optimal levels of 1,320 and 1,404 FYT/kg during nursery and growing–finishing phases, respectively. Carcass traits were also affected by dietary treatments (p < 0.05). Pigs fed the NC diet showed lower final body weight (FBW), hot carcass weight (HCW), carcass yield (CY), and lean meat percentage (LMP) compared with PC, whereas phytase supplementation restored these variables to values comparable to PC. Quadratic responses to phytase inclusion were observed for FBW (p = 0.004), HCW (p < 0.001), CY (p = 0.028), and LMP (p < 0.001), with estimated optimal inclusion levels of 1,377, 1,324, 1,416, and 1,246 FYT/kg, respectively. In conclusion, phytase supplementation effectively mitigated the negative effects of dietary Ca and P restriction, maintaining growth performance and carcass characteristics comparable to a nutritionally adequate diet across all production phases.

## Introduction

Phytases are the most widely used exogenous enzymes in monogastric nutrition, primarily due to their capacity to hydrolyze phytate-bound phosphorus present in plant-based feed ingredients [1]. Most commercial phytases are produced from bacterial sources and developed through genetic engineering, including enzymes derived from *Escherichia coli* or *Citrobacter braakii* and expressed in fungal hosts such as *Trichoderma reesei* or *Aspergillus oryzae* [2–4]. Based on the position of the initial orthophosphate cleavage on the phytate molecule, phytases are classified as 3- or 6-phytases, a distinction that directly influences their catalytic properties, including optimal pH range, hydrolytic efficiency, and thermal and gastric stability [5–7].

The nutritional benefits of phytase supplementation in monogastric diets are well established and primarily involve enhanced utilization of phytate-bound phosphorus, resulting in reduced antinutritional effects and lower phosphorus excretion into the environment [3]. In addition to phosphorus release, phytase-mediated phytate degradation increases the availability of myo-inositol and disrupts phytate–nutrient complexes, which would otherwise impair mineral absorption and increase endogenous nutrient losses [8,9]. These mechanisms support the so-called extra-phosphoric effects of phytases, which include improvements in the digestibility of calcium, amino acids, protein, and dietary energy, thereby contributing to enhanced feed efficiency and sustainability in swine production systems [10,11].

Most phytases currently used in commercial practice are classified as third-generation additives and represent a substantial improvement over earlier enzyme technologies developed in the late 1980s and early 1990s [12]. Nevertheless, continuous advances in protein engineering have led to the development of fourth-generation phytases, such as those derived from *Citrobacter braakii* and expressed in *Aspergillus oryzae*, which exhibit higher intrinsic activity, improved thermal resistance, and broader pH tolerance [4].

Despite the widespread use of phytases, most studies have focused on third-generation enzymes and moderate reductions in dietary calcium and phosphorus. Information regarding the efficacy of fourth-generation phytases, particularly under conditions of severe mineral restriction and across the entire production cycle, remains limited. Understanding the dose–response relationship of these enzymes is relevant to optimize their application and to define the level at which additional supplementation no longer results in biological benefits. Therefore, the objective of the present study was to evaluate the effects of increasing doses of a novel fourth-generation phytase on growth performance and carcass characteristics of pigs from nursery to finishing phases. To challenge enzyme efficacy and characterize dose–response responses, pigs were fed corn–soybean meal–based diets with reduced calcium and available phosphorus levels, representing a nutritional scenario more stringent than those commonly evaluated in previous studies.

## Materials and methods

The study was conducted in accordance with the guidelines of the Brazilian National Council for the Control of Animal Experimentation (CONCEA) for the Care and Use of Laboratory Animals and was approved by the Animal Ethics Committee of Akei Animal Research (protocol number 004/21). A total of 250 weaned piglets (AG337 × Camborough^®^), comprising 125 females and 125 castrated males, with an initial body weight of 6.079 ± 0.744 kg and 21 days of age, were used in the experiment. Animals were allocated in a randomized block design, based on initial body weight, with five dietary treatments and ten replicates per treatment. Each pen housed five pigs and was considered the experimental unit.

Pigs were housed in a commercial research facility with environmentally controlled pens equipped with nipple drinkers and feeders, allowing ad libitum access to feed and water throughout the study. During the experimental period, the average air temperature and relative humidity were 24.5 ± 4.9 °C and 64.8 ± 12.7%, respectively.

The treatments consisted of five experimental diets: a positive control (PC), supplemented with inorganic phosphorus (P) and calcium (Ca) provided by dicalcium phosphate and limestone; a negative control (NC), formulated with a reduction in available phosphorus (−0.18%) and calcium (−0.16%); and three NC diets supplemented with increasing levels of phytase at 600 (600 FYT), 1,200 (1,200 FYT), and 1,800 FYT/kg of feed. The phytase used was a bacterial 6-phytase (HiPhorius^®^, DSM Nutritional Products, Brazil), derived from *Citrobacter braakii* and expressed in *Aspergillus oryzae*, with declared activity in phytase units (FYT), defined as the amount of enzyme required to release 1 μmol of inorganic phosphorus per minute from sodium phytate under standard assay conditions.

Animals were subjected to an eight-phase feeding program: pre-starter I (21–28 days of age), pre-starter II (29–35 days), starter I (36–49 days), starter II (50–63 days), grower I (64–91 days), grower II (92–112 days), finisher I (113–133 days), and finisher II (134–156 days of age) (Tables 1 and 2). All diets were based on corn and soybean meal and were formulated to meet the minimum nutritional requirements recommended by Rostagno et al. [13], except for the deliberate reductions in calcium and available phosphorus in the NC diets. Feed and water were provided *ad libitum* throughout the experimental period. Average daily gain (ADG), average daily feed intake (ADFI), and feed conversion ratio (FCR) were evaluated at the beginning of the study and at the end of each feeding phase, corresponding to 28, 36, 49, 63, 91, 112, 140, and 156 days of age.

**Table 1:** Ingredient composition and calculated nutritional and energy values of experimental nursery diets for positive control (PC) and negative control (NC) treatments.

Ingredients (g/kg)	Pre-starter I		Pre-starter II		Starter I		Starter II	
	PC	NC	PC	NC	PC	NC	PC	NC
Ground corn (7.5%)	538.19	551.44	561.69	574.31	596.13	609.39	645.65	658.85
Soybean meal (45%)	206.00	206.00	248.00	248.00	280.00	280.00	289.00	289.00
Whey powder	150.00	150.00	100.00	100.00	50.00	50.00	0.00	0.00
Spray-dried plasma	50.00	50.00	25.00	25.00	0.00	0.00	0.00	0.00
Soybean oil	16.70	11.30	22.00	16.80	26.20	20.76	23.80	18.40
Dicalcium phosphate (24/18)	19.25	9.20	18.57	9.62	19.60	9.53	18.45	8.40
Limestone (36% Ca)	2.80	5.00	4.10	5.63	4.37	6.62	4.68	6.93
Salt	1.90	1.90	4.50	4.50	5.85	5.85	5.80	5.80
Zinc oxide (72%)	3.50	3.50	3.50	3.50	3.50	3.50	0.00	0.00
Copper sulfate (pentahydrate)	0.50	0.50	0.50	0.50	0.50	0.50	0.50	0.50
DL-methionine	1.70	1.70	1.75	1.75	1.89	1.89	1.67	1.67
L-lysine HCl	4.45	4.45	4.91	4.91	5.66	5.66	5.18	5.18
L-threonine	2.00	2.00	2.25	2.25	2.66	2.66	2.13	2.13
L-tryptophan	0.44	0.44	0.47	0.47	0.55	0.55	0.45	0.45
L-valine	0.82	0.82	1.01	1.01	1.34	1.34	0.94	0.94
Mycotoxin adsorbent^1^	1.50	1.50	1.50	1.50	1.50	1.50	1.50	1.50
Mineral premix^2^	0.10	0.10	0.10	0.10	0.10	0.10	0.10	0.10
Vitamin premix^3^	0.15	0.15	0.15	0.15	0.15	0.15	0.15	0.15
Nutrients and energy								
ME, kcal/kg	3350	3350	3350	3350	3350	3350	3350	3350
Crude protein, %	20.06	20.17	19.89	19.99	19.42	19.53	19.51	19.64
SID lysine, %	1.40	1.40	1.35	1.35	1.30	1.30	1.25	1.25
Calcium, %	0.75	0.59	0.75	0.59	0.75	0.59	0.70	0.54
Total phosphorus, %	0.71	0.54	0.68	0.52	0.68	0.50	0.64	0.46
Available phosphorus, %	0.55	0.37	0.50	0.34	0.48	0.30	0.43	0.25
Sodium, %	0.35	0.35	0.35	0.35	0.30	0.30	0.25	0.25
SID Met+Cys: SID Lys	0.56	0.56	0.56	0.56	0.56	0.56	0.57	0.57
SID Thr: SID Lys	0.67	0.67	0.67	0.67	0.67	0.67	0.65	0.65
SID Trp: SID Lys	0.19	0.19	0.19	0.19	0.19	0.19	0.19	0.19
SID Val: SID Lys	0.69	0.69	0.69	0.69	0.69	0.69	0.69	0.69
SID Ile: SID Lys	0.50	0.50	0.52	0.52	0.53	0.53	0.55	0.55

PC = positive control; NC = negative control; SID = standardized ileal digestibility. ^1^Mycotoxin adsorbent; ^2^Mineral premix provided per kg of diet: 100 mg Fe; 10 mg Cu; 40 mg Mn; 1 mg Co; 100 mg Zn; 1.5 mg I. ^3^Vitamin premix provided per kg of diet: 6,000 IU vitamin A; 1,500 IU vitamin D_3_; 15 mg vitamin E; 1.5 mg vitamin K_3_; 1.35 mg vitamin B_1_; 4 mg vitamin B_2_; 2 mg vitamin B_6_; 20 μg vitamin B_12_; 20 mg niacin; 9.35 mg pantothenic acid; 600 μg folic acid; 80 μg biotin; 300 μg Se.

**Table 2:** Ingredient composition and calculated nutritional and energy values of experimental growing and finishing diets for positive control (PC) and negative control (NC) treatments.

Ingredients (g/kg)	Grower I		Grower II		Finisher I		Finisher II	
	PC	NC	PC	NC	PC	NC	PC	NC
Ground corn (7.5%)	706.55	719.45	748.22	761.22	797.63	810.89	838.73	851.43
Soybean meal (45%)	235.00	235.00	199.00	199.00	161.00	161.00	125.00	125.00
Soybean oil	20.00	15.00	17.50	12.20	9.40	4.00	7.00	1.75
Dicalcium phosphate (24/18)	14.80	4.70	12.10	2.10	10.58	0.52	9.05	0.00
Limestone (36% Ca)	7.50	9.70	8.00	10.30	7.90	10.10	7.70	9.30
Salt	4.60	4.60	4.60	4.60	3.80	3.80	3.80	3.80
Copper sulfate (pentahydrate)	0.50	0.50	0.50	0.50	0.50	0.50	0.50	0.50
DL-methionine	1.43	1.43	1.11	1.11	0.86	0.86	0.52	0.52
L-lysine HCl	4.82	4.82	4.58	4.58	4.35	4.35	4.11	4.11
L-threonine	1.82	1.82	1.61	1.61	1.40	1.40	1.20	1.20
L-tryptophan	0.54	0.54	0.51	0.51	0.48	0.48	0.46	0.46
L-valine	0.69	0.69	0.52	0.52	0.35	0.35	0.18	0.18
Mycotoxin adsorbent^1^	1.50	1.50	1.50	1.50	1.50	1.50	1.50	1.50
Mineral premix^2^	0.10	0.10	0.10	0.10	0.10	0.10	0.10	0.10
Vitamin premix^3^	0.15	0.15	0.15	0.15	0.15	0.15	0.15	0.15
Nutrients and energy								
ME, kcal/kg	3350	3350	3350	3350	3325	3325	3325	3325
Crude protein, %	17.54	17.66	16.21	16.34	14.90	15.00	13.56	13.66
SID lysine, %	1.10	1.10	1.00	1.00	0.90	0.90	0.80	0.80
Calcium, %	0.70	0.54	0.65	0.49	0.60	0.44	0.55	0.39
Total phosphorus, %	0.56	0.38	0.51	0.33	0.47	0.29	0.43	0.27
Available phosphorus, %	0.36	0.18	0.31	0.13	0.28	0.10	0.25	0.09
Sodium, %	0.20	0.20	0.20	0.20	0.18	0.18	0.17	0.17
SID Met+Cys: SID Lys	0.59	0.59	0.59	0.59	0.60	0.60	0.60	0.60
SID Thr: SID Lys	0.65	0.65	0.65	0.65	0.65	0.65	0.65	0.65
SID Trp: SID Lys	0.20	0.20	0.20	0.20	0.20	0.20	0.20	0.20
SID Val: SID Lys	0.69	0.69	0.69	0.69	0.69	0.69	0.69	0.69
SID Ile: SID Lys	0.55	0.55	0.55	0.55	0.55	0.55	0.55	0.55

PC = positive control; NC = negative control; SID = standardized ileal digestibility. ^1^Mycotoxin adsorbent; ^2^Mineral premix provided per kg of diet: 100 mg Fe; 10 mg Cu; 40 mg Mn; 1 mg Co; 100 mg Zn; 1.5 mg I. ^3^Vitamin premix provided per kg of diet: 6,000 IU vitamin A; 1,500 IU vitamin D_3_; 15 mg vitamin E; 1.5 mg vitamin K_3_; 1.35 mg vitamin B_1_; 4 mg vitamin B_2_; 2 mg vitamin B_6_; 20 μg vitamin B_12_; 20 mg niacin; 9.35 mg pantothenic acid; 600 μg folic acid; 80 μg biotin; 300 μg Se.

At 156 days of age, all animals were transported to a commercial slaughterhouse. Prior to transport, pigs were subjected to a 12-h feed withdrawal period, while water remained available until slaughter. Animals were rendered unconscious by electrical stunning, followed by exsanguination through severing of the blood vessels in the neck region.

Carcasses were evaluated using an automated electronic carcass grading system (e.g., Hennessy Grading Probe, Hennessy Grading Systems, New Zealand), which measured backfat thickness (BF) and the depth of the Longissimus thoracis et lumborum muscle (LM depth) at the P2 site (59 mm lateral to the dorsal midline of the carcass, immediately caudal to the last rib, on the left half-carcass). Hot carcass weight (HCW) was recorded, and both the percentage and total amount of lean meat in the carcass were determined. Lean meat percentage was calculated using the modified equation:


$$\begin{array}{c} \text{Lean\textasciitilde{}meat\textasciitilde{}(\textbackslash{}\%)\textasciitilde{}}=\text{\textasciitilde{}}61.33-\left[0.76\times BF\right]+\left[0.10\times LM\text{\textasciitilde{}depth}\right] \end{array}$$

and total lean meat content was obtained by multiplying carcass weight by the corresponding lean percentage.

Data were initially screened for outliers using the interquartile range (IQR) method with a 1.5 coefficient, and identified values were excluded prior to analysis. Normality of residuals and homogeneity of variances were evaluated using the Shapiro–Wilk test and Levene’s test, respectively (p > 0.05). Model assumptions were verified through residual analysis. Each pen was considered the experimental unit for growth performance variables, whereas individual animals were considered the experimental unit for carcass characteristics. Data were analyzed using analysis of variance (ANOVA) within the General Linear Model (GLM) framework, considering dietary treatment as a fixed effect. Block effects based on initial body weight were included in the model. To characterize the response to increasing phytase inclusion levels, linear and quadratic polynomial contrasts were applied, allowing the identification of dose–response patterns and estimation of optimal phytase inclusion levels for performance and carcass traits. When significant quadratic effects were detected, regression equations were used to estimate the phytase level associated with the maximal biological response. Treatment means were compared using Tukey’s test for pairwise comparisons. Differences were considered statistically significant at p ≤ 0.05, whereas values between 0.05 < p ≤ 0.10 were interpreted as statistical trends. All statistical analyses were performed using Statistica^®^ for Windows, version 10.0 (StatSoft Inc., Tulsa, OK, USA) [14].

## Results

During the pre-starter I and II phases (21–35 d), no effects of dietary treatments were observed on initial body weight (IBW), ADFI, ADG, FCR, or final body weight (FBW) (Table 3; p > 0.05), and no linear or quadratic responses to increasing phytase levels were detected (p > 0.05).

**Table 3:** Mean initial body weight (IBW), average daily gain (ADG), average daily feed intake (ADFI), feed conversion ratio (FCR), and final body weight (FBW) of pigs during each nursery phase according to experimental treatment.

Phase / Variable	Experimental groups					CV (%)	p-value		
	PC	NC	600 FYT	1,200 FYT	1,800 FYT		ANO	Lin^R^	Quad^R^
Pre-starter I (21–28 days)									
IBW (kg)	6.08	6.078	6.079	6.081	6.079	12.1	1.000	NS	NS
ADFI (kg)	0.200	0.185	0.182	0.156	0.184	22.7	0.440	NS	NS
ADG (kg)	0.170	0.145	0.161	0.128	0.137	34.8	0.441	NS	NS
FCR	1.379	1.304	1.175	1.196	1.387	26.2	0.281	NS	NS
FBW (kg)	7.273	7.093	7.205	6.979	7.035	12.0	0.956	NS	NS
Pre-starter II (29–35 days)									
ADFI (kg)	0.445	0.439	0.445	0.400	0.408	14.2	0.277	NS	NS
ADG (kg)	0.340	0.306	0.334	0.294	0.304	14.4	0.167	NS	NS
FCR	1.317	1.435	1.331	1.365	1.350	9.3	0.245	NS	NS
FBW (kg)	9.653	9.290	9.545	9.036	9.164	11.1	0.755	NS	NS
Starter I (36–49 days)									
ADFI (kg)	0.751	0.701	0.697	0.663	0.643	11.4	0.243	0.036^a^	NS
ADG (kg)	0.489^a^	0.365^c^	0.418^bc^	0.441^ab^	0.393^bc^	16.1	0.000	NS	NS
FCR	1.553^bc^	1.924^a^	1.671^b^	1.509^c^	1.641^b^	10.3	0.000	NS	<0.001^b^
FBW (kg)	16.592	14.407	15.393	15.208	14.660	10.9	0.059	NS	NS
Starter II (50–63 days)									
ADFI (kg)	1.116	0.986b	1.027	1.041	1.030	9.3	0.083	NS	NS
ADG (kg)	0.698	0.602	0.652	0.664	0.646	11.6	0.051	NS	NS
FCR	1.601	1.643	1.589	1.577	1.601	6.9	0.815	0.022^c^	NS
FBW (kg)	26.360^a^	22.838^b^	24.132^ab^	24.587^ab^	24.048^ab^	10.1	0.023	NS	NS
Total phase (21–63 days)									
ADFI (kg)	1.116^a^	0.986^b^	1.083^ab^	1.095^ab^	1.030^ab^	9.3	0.033	NS	NS
ADG (kg)	0.698^a^	0.602^b^	0.701^a^	0.693^ab^	0.680^ab^	11.2	0.019	NS	NS
FCR	1.601^ab^	1.643^a^	1.548^ab^	1.587^ab^	1.519^b^	6.3	0.048	NS	<0.001^d^

PC = Positive control. NC = Negative control. 600FYT = NC + 600 FYT/kg of feed. 1,200FYT = NC + 1,200 FYT/kg of feed. 1,800FYT = NC + 1,800 FYT/kg of feed. FYT = phytase units per kilogram of feed. CV = coefficient of variation. NS = not significant. ANO = p-value from analysis of variance (ANOVA). Lin^R^ = p-value for linear regression. Quad^R^ = p-value for quadratic regression. Means followed by different letters within the same row differ significantly according to Tukey’s test (p ≤ 0.05). p-values refer to linear and quadratic responses to increasing dietary phytase levels. ^a^ Y = 0.7075 − 0.000034729X; p = 0.0367; R^2^ = 0.1099, ^b^ Y = 1.9344 − 0.0006X + 0.00000026736X^2^; p < 0.001; R^2^ = 0.0468, ^c^ Y = 1.6244 − 0.000055398X; p = 0.0227; R^2^ = 0.1326, ^d^ Y = 1.671 − 0.0003X + 0.00000010526X^2^; p < 0.001; R^2^ = 0.4092.

In the starter I phase (36–49 d), dietary treatments affected ADG and FCR (p < 0.001), whereas ADFI remained unchanged (p = 0.243). Pigs fed the NC diet exhibited a 25.35% reduction in ADG compared with the PC group (0.365 vs. 0.489 kg/d), and a 17.23% lower ADG compared with pigs receiving 1,200 FYT. Feed efficiency was impaired in the NC group, with FCR worsening by 23.89% relative to PC (1.924 vs. 1.553). In contrast, supplementation with 1,200 FYT improved FCR by 21.57% compared with NC. Within phytase-supplemented treatments, ADFI increased linearly (p = 0.036), and FCR showed a quadratic response (p < 0.001), with an estimated optimum at 1,114 FYT/kg.

In the starter II phase (50–63 d), only FBW was affected by dietary treatments (p = 0.023). Pigs fed the PC diet showed a 13.36% higher FBW compared with NC (26.36 vs. 22.84 kg), whereas phytase-supplemented groups presented intermediate values, approaching those of PC. No treatment effects were observed for ADFI or ADG (p > 0.05). However, FCR decreased linearly with increasing phytase inclusion (p = 0.022), indicating improved feed efficiency.

Considering the overall nursery period (21–63 d), dietary treatments affected ADFI (p = 0.033), ADG (p = 0.019), and FCR (p = 0.048). The NC group showed reductions of 11.64% in ADFI and 13.75% in ADG compared with PC, whereas phytase supplementation restored these parameters to levels comparable to the PC group. Feed conversion ratio exhibited both linear and quadratic responses to increasing phytase levels (p < 0.001), with an estimated optimum at 1,320 FYT/kg.

During the grower I phase (64–91 d), pigs fed the NC diet exhibited impaired performance compared with all other treatments. ADFI was reduced by 20.9% relative to PC (1.590 vs. 1.922 kg/d), while ADG decreased by 23.0% (p < 0.001). Feed efficiency was also negatively affected, with the highest FCR observed in the NC group (2.060). Final body weight was 22.0% lower in NC pigs compared with PC (44.43 vs. 54.39 kg. Quadratic responses to increasing phytase inclusion were observed for ADFI, ADG, FCR, and FBW (p < 0.05), with estimated optima at 1,263, 1,219, 1,280, and 1,116 FYT/kg, respectively.

In the grower II phase (92–112 d), the negative impact of the NC diet persisted. ADFI and ADG were reduced by 18.8% and 22.1%, respectively, compared with PC (p < 0.001). Feed conversion ratio was impaired in NC pigs, whereas supplementation with 1,800 FYT/kg improved FCR by 9.4% relative to NC. Final body weight remained lower in the NC group compared with all other treatments. Dose–response analysis revealed quadratic effects for ADG and FCR (p < 0.001), with estimated optima at 1,540 and 1,217 FYT/kg, respectively, while FBW increased linearly with phytase inclusion.

During the finisher I phase (113–133 d), pigs fed the PC and phytase-supplemented diets showed higher ADFI, ADG, and FBW compared with NC pigs (p < 0.001). Average daily gain in NC pigs was reduced by 24.1% relative to PC (0.880 vs. 1.159 kg/d), whereas phytase-supplemented pigs maintained growth performance comparable to PC. Feed conversion ratio was poorest in NC pigs, while supplementation with 600 and 1,800 FYT/kg improved feed efficiency. Quadratic responses to phytase inclusion were observed for all performance variables (p < 0.01), with estimated optima ranging from 1,361 to 1,608 FYT/kg.

In the finisher II phase (134–156 d), NC pigs again showed reduced performance. ADG was 28.7% lower than in PC pigs (0.685 vs. 0.961 kg/d), and FBW was reduced by 21.7% (95.6 vs. 122.1 kg). Phytase supplementation restored ADG and FBW to levels comparable to PC. Quadratic dose–response relationships were observed for ADFI, ADG, FCR, and FBW (p < 0.05), with estimated optimal inclusion levels ranging from 1,193 to 1,778 FYT/kg.

Considering the overall growing and finishing period (64–156 d), pigs fed phytase-supplemented diets showed higher ADFI, ADG, and improved FCR compared with NC (p < 0.05), while maintaining performance comparable to PC. Relative to NC, ADG increased by approximately 29.8%, 30.6%, and 31.0% in pigs receiving 600, 1,200, and 1,800 FYT/kg, respectively. ADFI and FCR exhibited quadratic responses to phytase inclusion (p < 0.01), with estimated optima at 1,296 and 1,404 FYT/kg, respectively, whereas ADG increased linearly with increasing phytase levels.

Considering the overall experimental period (21–156 d), dietary treatments affected ADFI, ADG, and FCR (p < 0.001). Pigs fed the NC diet showed a 17.7% reduction in ADFI compared with PC pigs (1.674 vs. 2.034 kg/d). In contrast, phytase supplementation restored ADFI to levels comparable to PC, with values ranging from 1.922 to 1.972 kg/d. A quadratic response to increasing phytase inclusion was observed for ADFI (p < 0.001).

Average daily gain followed a similar pattern. Pigs fed the NC diet exhibited a 23.1% reduction in ADG compared with PC (0.667 vs. 0.867 kg/d). Phytase supplementation restored growth performance, with ADG values of 0.832, 0.845, and 0.854 kg/d for pigs receiving 600, 1,200, and 1,800 FYT/kg, respectively, all comparable to the PC group. A quadratic response of ADG to increasing phytase inclusion was detected (p < 0.001), indicating a dose-dependent improvement in growth rate.

Feed conversion ratio was also affected by dietary treatments. Pigs fed the NC diet exhibited poorer feed efficiency than PC pigs, with FCR worsening by 7.1% (2.512 vs. 2.346). In contrast, phytase-supplemented pigs showed improved feed efficiency, with FCR values ranging from 2.299 to 2.309, all lower than those observed in NC pigs. Feed conversion ratio decreased linearly with increasing phytase inclusion (p < 0.001).

Regarding carcass traits (Table 6), dietary treatments affected FBW, HCW, CY, LMD, and LMP (p < 0.05), whereas no differences were observed for backfat thickness (BF) (p = 0.558). Pigs fed the NC diet showed inferior carcass traits compared with PC and phytase-supplemented pigs, while no differences were detected between PC and phytase treatments for most variables. Final body weight was reduced by 16.9% in NC pigs compared with PC (101.32 vs. 122.02 kg; p < 0.001). In contrast, phytase supplementation restored FBW to levels comparable to PC, ranging from 118.91 to 120.34 kg. A quadratic response to increasing phytase inclusion was observed (p = 0.004), with an estimated optimum at approximately 1,251 FYT/kg.

Hot carcass weight followed a similar pattern, with NC pigs showing a 20.3% reduction compared with PC (69.41 vs. 87.03 kg; p < 0.001). Phytase supplementation restored HCW to values comparable to PC (84.01–84.53 kg), with a quadratic response (p < 0.001) and estimated optimum at 1,250 FYT/kg. Carcass yield was also reduced in NC pigs compared with PC (68.62 vs. 71.38%; −3.9%; p = 0.030), and exhibited a quadratic response to phytase inclusion (p = 0.028), with an estimated optimum at 1,240 FYT/kg.

In contrast, longissimus muscle depth (LMD) was reduced by 10.3% in NC pigs compared with PC (57.59 vs. 64.18 mm; p < 0.001). Phytase-supplemented pigs showed intermediate values, with the 1,200 FYT treatment reaching levels comparable to PC. Lean meat traits were also negatively affected by the NC diet. Lean meat weight (kg) was reduced by 4.3% in NC pigs compared with PC (53.66 vs. 56.06 kg; p = 0.004). Similarly, lean meat percentage (%) was markedly reduced by 21.3% in NC pigs relative to PC (37.97 vs. 48.25%; p < 0.001). A quadratic response of lean meat percentage to increasing phytase inclusion was observed (p < 0.001), with an estimated optimum at approximately 1,200 FYT/kg

## Discussion

During the pre-starter I and II phases, the absence of treatment effects on growth performance indicates that the reduction of dietary Ca and P without phytase supplementation did not limit piglet performance under the experimental conditions (Table 3). This response is likely associated with the low voluntary feed intake and unstable growth dynamics typically observed immediately after weaning, which reduce short-term mineral demand and may mask dietary deficiencies [15].

From the starter I phase onward, however, the negative effects of mineral restriction became evident, as previously demonstrated in the results, with clear reductions in ADG and feed efficiency in NC pigs. Phytase supplementation effectively reversed these impairments, restoring performance to levels comparable to the PC group. This temporal response supports the concept that mineral inadequacy becomes limiting once feed intake and growth rate increase, reaching a threshold at which Ca and P supply constrains tissue accretion and nutrient utilization efficiency [16,17].

This pattern can be partially explained by physiological and enzymatic factors influencing phytase efficacy. Phytase activity is strongly dependent on gastrointestinal pH, and differences among enzyme sources in their optimal pH range directly affect their *in vivo* functionality [3]. Early post-weaning piglets exhibit relatively high and variable gastric pH (approximately 4–5, with regional variation), which can limit the activity of acid-optimized enzymes and reduce phytate hydrolysis during this period [18–20]. This physiological condition provides a plausible explanation for the lack of performance differences observed in the earliest phases, despite the imposed mineral restriction.

As pigs mature, gastric acid secretion increases and luminal pH decreases, creating a more favorable environment for phytase activity. In this context, fourth-generation phytases, such as the one evaluated in the present study, are expected to express greater efficacy in later growth phases, when gastrointestinal conditions better match their activity profile [18,20,21]. This interpretation is consistent with the observed recovery in performance from the starter phase onward and aligns with evidence that these enzymes exhibit enhanced stability and activity across a broader pH range compared with earlier phytase generations restriction [4,20–22].

Adult pigs regulate gastric pH through hydrochloric acid secretion, whereas newly weaned piglets exhibit higher and more variable gastric pH values [18]. Gastric acid secretion capacity increases progressively with age and feed intake, resulting in a more acidic and stable gastric environment in growing and finishing pigs [23]. Given that enhanced stability under low pH is a key feature of fourth-generation phytases, their efficacy is expected to increase as gastrointestinal conditions mature, which is consistent with the performance responses observed in the present study across later production phases [18,24].

Phytase efficacy is influenced by multiple interacting factors, including enzyme origin, molecular structure, resistance to proteolysis, animal age, digesta retention time, dietary phytate concentration, calcium levels, and overall diet composition [3]. Despite this complexity, phytase supplementation consistently supported growth performance comparable to the PC group and superior to the NC treatment across all phases evaluated. This consistent response indicates that the enzymatic activity was sufficient to overcome the imposed Ca and P restriction under a wide range of physiological conditions, reinforcing its robustness under practical feeding scenarios [4].

Regarding phytase superdosing, the inclusion levels of 1,200 and 1,800 FYT/kg evaluated in this study fall within the range typically associated with superdosing strategies. These approaches extend beyond phosphorus release, promoting more complete phytate degradation and improving nutrient utilization, including amino acids and energy [8,9,25]. In the present study, optimal growth responses were consistently observed within the range of approximately 1,200–1,700 FYT/kg across production phases (Tables 3–5), supporting the concept of a biological optimum rather than a maximal inclusion rate [26,27].

**Table 4:** Mean initial body weight (IBW), average daily feed intake (ADFI), average daily gain (ADG), feed conversion ratio (FCR), and final body weight (FBW) of pigs during the growth and finishing period according to experimental treatment.

Phase / Variable	Experimental groups					CV (%)	p-value		
	PC	NC	600 FYT	1,200 FYT	1,800 FYT		ANO	Lin^R^	Quad^R^
Growth I (64–91 days)									
IBW (kg)	26.360^a^	22.838^b^	25.209^ab^	24.913^ab^	24.176^ab^	9.8	0.015	NS	NS
ADFI (kg)	1.922^a^	1.590^b^	1.797^ab^	1.799^ab^	1.767^ab^	10.9	0.017	NS	0.041^a^
ADG (kg)	1.001^a^	0.771^b^	0.966^a^	0.963^a^	0.946^a^	11.9	<0.001	NS	<0.001^b^
FCR	1.918^b^	2.060^a^	1.860^b^	1.869^b^	1.868^b^	5.4	<0.001	NS	<0.001^c^
FBW (kg)	54.393^a^	44.434^b^	52.263^a^	51.870^a^	50.656^a^	10.0	<0.001	NS	0.009^d^
Growth II (92–112 days)									
ADFI (kg)	2.535^a^	2.060^b^	2.482^a^	2.427^a^	2.409^a^	11.3	<0.001	NS	0.009^e^
ADG (kg)	1.060^a^	0.826^b^	1.018^a^	1.046^a^	1.063^a^	12.2	<0.001	NS	<0.001^f^
FCR	2.396^ab^	2.502^a^	2.448^ab^	2.318^ab^	2.266^b^	7.0	0.010	<0.001^g^	NS
FBW (kg)	76.644^a^	61.780^b^	73.630^a^	73.837^a^	72.978^a^	9.8	<0.001	<0.001^h^	NS
Finishing I (113–133 days)									
ADFI (kg)	3.135^a^	2.544^b^	3.111^a^	3.168^a^	3.136^a^	12.9	0.001	NS	<0.001^i^
ADG (kg)	1.159^a^	0.880^b^	1.195^a^	1.149^a^	1.218^a^	14.4	<0.001	NS	<0.001^j^
FCR	2.707^ab^	2.899^a^	2.603^b^	2.760^ab^	2.575^b^	7.1	0.001	NS	0.002^k^
FBW (kg)	100.977^a^	80.261^b^	98.734^a^	97.959^a^	98.558^a^	10.1	<0.001	NS	<0.001^l^
Finishing II (134–156 days)									
ADFI (kg)	3.107	2.491	2.938	2.768	3.015	15.6	0.057	NS	0.032^m^
ADG (kg)	0.961^a^	0.685^b^	0.910^a^	0.983^a^	0.937^a^	17.5	0.003	NS	<0.001^n^
FCR	3.237	3.698	3.241	3.132	3.222	11.1	0.066	NS	0.005^o^
FBW (kg)	122.127^a^	95.605^b^	118.808^a^	119.542^a^	119.204^a^	10.6	<0.001	NS	<0.001^p^
Overall growth and finishing (64–156 days)									
ADFI (kg)	2.623^a^	2.130^b^	2.527^a^	2.554^a^	2.524^a^	11.0	0.003	NS	0.001^q^
ADG (kg)	1.041^a^	0.788^b^	1.017^a^	1.029^a^	1.032^a^	12.1	<0.001	<0.001^r^	NS
FCR	2.519^b^	2.705^a^	2.483^b^	2.481^b^	2.443^b^	5.3	0.002	NS	<0.001^s^

PC = positive control; NC = negative control. 600 FYT = NC + 600 FYT/kg; 1,200 FYT = NC + 1,200 FYT/kg; 1,800 FYT = NC + 1,800 FYT/kg. FYT = phytase units per kg of feed. CV = coefficient of variation. NS = not significant. ANO = p-value from analysis of variance (ANOVA). Lin^R^ = p-value for linear regression. Quad^R^ = p-value for quadratic regression. Means within a row followed by different superscript letters differ significantly according to Tukey’s test (p < 0.05). p-values indicate linear and quadratic responses to increasing dietary phytase levels. ^a^ Y = 1.5982 + 0.0004X − 0.00000016607X^2^; p = 0.0413; R^2^ = 0.2237, ^b^ Y = 0.7805 + 0.0004X − 0.0000001472X^2^; p = 0.0007; R^2^ = 0.5055, ^c^ Y = 2.0488 − 0.0003X + 0.00000013821X^2^; p = 0.0001; R^2^ = 0.5822, ^d^ Y = 44.804 + 0.0143X − 0.0000062795X^2^; p = 0.0096; R^2^ = 0.3797, ^e^ Y = 2.0858 + 0.0007X − 0.00000030541X^2^; p = 0.0097; R^2^ = 0.3357, ^f^ Y = 0.8335 + 0.0003X − 0.00000012125X^2^; p < 0.001; R^2^ = 0.4182, ^g^ Y = 2.5094 − 0.0001X; p = 0.0002; R^2^ = 0.3085, ^h^ Y = 65.4715 + 0.0056X; p = 0.0004; R^2^ = 0.2933, ^i^ Y = 2.5652 + 0.0011X − 0.00000041572X^2^; p = 0.0009; R^2^ = 0.4055, ^j^ Y = 0.904 + 0.0005X − 0.00000017078X^2^; p < 0.001; R^2^ = 0.5259, ^k^ Y = 2.8589 − 0.0003X + 0.000000077271X^2^; p = 0.0026; R^2^ = 0.2350, ^l^ Y = 81.2922 + 0.0314X − 0.000012412X^2^; p < 0.001; R^2^ = 0.5831, ^m^ Y = 2.5431 + 0.0005X − 0.00000013829X^2^; p = 0.0321; R^2^ = 0.1326, ^n^ Y = 0.6865 + 0.0005X − 0.00000018825X^2^; p = 0.0003; R^2^ = 0.4741, ^o^ Y = 3.6906 − 0.0009X + 0.00000037947X^2^; p = 0.0051; R^2^ = 0.3215, ^p^ Y = 96.6745 + 0.0413X − 0.000016348X^2^; p < 0.001; R^2^ = 0.6292, ^q^ Y = 2.1453 + 0.0007X − 0.00000029618X^2^; p = 0.0010; R^2^ = 0.4051, ^r^ Y = 0.8543 + 0.0001X; p < 0.001; R^2^ = 0.4449, ^s^ Y = 2.6924 − 0.0004X + 0.00000012768X^2^; p < 0.001; R^2^ = 0.5196.

**Table 5:** Average daily feed intake (ADFI), average daily gain (ADG), and feed conversion ratio (FCR) of pigs during the overall experimental period (nursery, growth, and finishing) according to experimental treatment.

Variable	Experimental groups					CV (%)	p-value		
	PC	NC	600 FYT	1,200 FYT	1,800 FYT		ANO	Lin^R^	Quad^R^
Overall experimental period (21–156 days)									
ADFI (kg)	2.034^a^	1.674^b^	1.922^a^	1.945^a^	1.972^a^	10.1	<0.001	NS	<0.001^a^
ADG (kg)	0.867^a^	0.667^b^	0.832^a^	0.845^a^	0.854^a^	10.9	<0.001	NS	<0.001^b^
FCR	2.346^b^	2.512^a^	2.308^b^	2.299^b^	2.309^b^	5.0	<0.001	<0.001^c^	NS

PC = positive control; NC = negative control; 600 FYT = NC + 600 FYT/kg; 1,200 FYT = NC + 1,200 FYT/kg; 1,800 FYT = NC + 1,800 FYT/kg. FYT = phytase units per kg of feed; CV = coefficient of variation; NS = not significant. ANO = p-value from analysis of variance (ANOVA). Lin^R^ = p-value for linear regression. Quad^R^ = p-value for quadratic regression. Means within a row followed by different superscript letters differ significantly according to Tukey’s test (p < 0.05). p-values indicate linear and quadratic responses to increasing dietary phytase levels. Regression equations: ^a^ Y = 1.686 + 0.0005X − 0.00000021552X^2^; p = 0.0001; R^2^ = 0.2281, ^b^ Y = 0.6746 + 0.0003X − 0.00000012406X^2^; p < 0.001; R^2^ = 0.4261, ^c^ Y = 2.4605 − 0.0001X; p < 0.001; R^2^ = 0.3907.

The present findings differ from those reported by Gonçalves, Dritz, and Tokach [28], who observed more pronounced responses to phytase superdosing during the nursery phase, with less consistent effects in later production stages. In contrast, the current study demonstrated consistent responses across all phases, particularly during growth and finishing. Such discrepancies are commonly reported in phytase dose–response studies and can be attributed to differences in enzyme source, molecular structure, catalytic efficiency, and stability under gastrointestinal conditions [24,26,29]. In this context, it is likely that the phytases evaluated in previous studies did not exhibit the same technological characteristics as fourth-generation phytases, such as enhanced activity under low pH and increased resistance to thermal and proteolytic challenges [4,12].

Additionally, optimal phytase inclusion levels reported in earlier studies typically ranged from 2,000–3,000 FYT/kg, which are substantially higher than those identified in the present study. This difference suggests improved enzymatic efficiency and biological response associated with newer phytase generations, allowing maximal responses to be achieved at lower inclusion levels [26,27].

Another important factor is the degree of dietary Ca and P restriction. Many previous studies adopted less severe mineral reductions than those applied in the present study [27,30–33]. The magnitude of mineral restriction is a key determinant of the observed response to phytase supplementation, as more pronounced deficiencies increase the likelihood of detecting improvements in performance and nutrient utilization [34]. For example, Holloway et al. [35], reported improved performance with phytase superdosing across phases; however, the greatest responses were associated with the highest enzyme inclusion levels. In contrast, in the present study, maximal biological responses were achieved at lower phytase doses under more severe Ca and P restriction, reinforcing that dietary context strongly modulates the apparent optimal phytase inclusion level [26].

The concept of phytase superdosing extends beyond improvements in Ca and P digestibility, encompassing enhanced availability of amino acids, energy, and other nutrients through more complete phytate degradation [8–10,25–27,31]. This broader mechanistic framework explains why phytase supplementation is frequently associated with improvements in growth performance and feed efficiency, particularly under nutritionally challenging conditions [3,10,29,36]. This interpretation is consistent with the present findings, in which phytase supplementation restored growth performance to levels comparable to the PC diet, with optimal inclusion levels consistently identified between 1,200 and 1,700 FYT/kg.

Regarding mineral digestibility, Almeida, Sulabo, and Stein [2] demonstrated a linear increase (p < 0.05) in Ca and P digestibility with increasing phytase inclusion levels in growing–finishing pigs. Similar linear and curvilinear responses have been widely reported in the literature, reinforcing the dose–response relationship between phytase supplementation and mineral utilization [24,37], which is consistent with the performance responses observed in the present study.

Similarly, Adedokun et al. [37] demonstrated that phytase supplementation (0–2,000 FYT/kg) improved phosphorus and calcium utilization, as well as ileal digestibility of nitrogen and amino acids, in nursery and growing pigs. These responses are consistent with the disruption of phytate–protein complexes and the reduction of endogenous nutrient losses [8,10,26]. The dose-dependent enzymatic response reported by these authors aligns with the present findings, particularly the linear increase in ADG during the growing–finishing period (Table 4) and the linear improvement in FCR over the entire experimental period (Table 5), reinforcing the biological relevance of incremental phytase inclusion [24,37].

Earlier studies, such as Braña et al. [7], also reported dose-dependent improvements in performance and nutrient digestibility when evaluating different phytases across increasing inclusion levels, including tolerance doses up to 10,000 FTU/kg. These findings further support the biological rationale for phytase superdosing and its association with progressive improvements in nutrient utilization [26,27].

The fourth-generation phytase evaluated in the present study is characterized by enhanced thermal stability and a broader pH activity spectrum [12]. Thermal stability is particularly relevant under commercial feed processing conditions, as pelleting temperatures can reduce enzyme recovery and post-pellet activity [38]. Likewise, an expanded pH activity range increases the likelihood of enzyme activity across gastrointestinal compartments, improving phytate hydrolysis efficiency [18,24]. Although studies in pigs remain limited, evidence from broiler chickens indicates that this phytase provides greater phytate degradation and phosphorus digestibility compared with third-generation phytases, supporting its enhanced enzymatic efficiency and dose responsiveness [3,12].

Finally, the performance reductions observed in NC pigs in the present study are consistent with previous reports demonstrating that phosphorus deficiency impairs growth performance, including reductions in ADG and ADFI [4]. These effects reflect the central role of phosphorus in energy metabolism, skeletal development, and lean tissue accretion [21], further supporting the biological plausibility of the responses observed in this study.

The performance impairment observed under the NC diet was progressively alleviated with increasing inclusion levels of the fourth-generation phytase, confirming the dose-dependent responses identified in the present study. These findings reinforce that phytase efficacy becomes more pronounced as nutritional constraints intensify, particularly under restricted Ca and P supply [3,39]. Notably, the severity of mineral reduction applied in this study (−0.16% Ca and −0.18% available P) exceeds that of most previous studies, highlighting the capacity of this phytase to sustain performance under highly challenging nutritional conditions [2,27,28,31].

Regarding carcass characteristics (Table 6), the present results are consistent with those reported Silva et al. [27,31], who observed that phytase supplementation does not increase fat deposition. This suggests that the additional nutrients released under phytase supplementation, particularly under superdosing conditions, are preferentially directed toward lean tissue accretion rather than adipose deposition [40]. This interpretation is supported by the absence of differences in backfat thickness and the marked recovery of lean-related traits observed in the present study.

**Table 6:** Final body weight and carcass traits of pigs fed diets with different phytase inclusion levels according to experimental treatment.

Variable	Experimental groups					CV (%)	p-value		
	PC	NC	600 FYT	1,200 FYT	1,800 FYT		ANO	Lin^R^	Quad^R^
FBW (Kg)	122.020^a^	101.323^b^	118.907^a^	120.343^a^	119.202^a^	10.0	<0.001	NS	0.004^a^
HCW (Kg)	87.031^a^	69.411^b^	84.007^a^	84.529^a^	84.010^a^	10.7	<0.001	NS	<0.001^b^
CY (%)	71.377^a^	68.619^b^	70.662^a^	70.253^a^	70.465^a^	3.6	0.030	NS	0.028^c^
BF (mm)	16.940	16.701	17.480	17.062	18.043	20.6	0.558	NS	NS
LMD (mm)	64.180^a^	57.585^c^	61.512^ab^	63.571^ab^	59.793^b^	10.9	0.001	NS	NS
LMP (Kg)	56.061^a^	53.662^b^	55.407^ab^	56.026^a^	54.760^ab^	4.4	0.004	NS	NS
LMP (%)	48.253^a^	37.969^b^	46.524^a^	47.286^a^	45.953^a^	9.9	<0.001	NS	<0.001^d^

PC = positive control; NC = negative control; 600 FYT = NC + 600 FYT/kg; 1,200 FYT = NC + 1,200 FYT/kg; 1,800 FYT = NC + 1,800 FYT/kg. FYT = phytase units per kg of feed; CV = coefficient of variation; NS = not significant. ANO = p-value from analysis of variance (ANOVA). LinR = p-value for linear regression. QuadR = p-value for quadratic regression. Means within a row followed by different superscript letters differ according to Tukey’s test (p < 0.05). p-values indicate linear and quadratic responses to increasing dietary phytase levels. Regression equations: ^a^ Y = 106.9641 + 0.0234X − 0.0000093515X^2^; p = 0.0040; R^2^ = 0.0595, ^b^ Y = 72.5615 + 0.0216X − 0.0000086399X^2^; p = 0.0006; R^2^ = 0.0838, ^c^ Y = 67.9048 + 0.0047X − 0.0000018944X^2^; p = 0.0285; R^2^ = 0.0348^d^ Y = 38.7895 + 0.0155X − 0.00000646X^2^; p = 0.0005; R^2^ = 0.0864.

Final body weight and hot carcass weight followed patterns similar to those described in the literature [31], although the optimal phytase inclusion levels identified in the present study (~1,300–1,400 FYT/kg) were substantially lower than those reported for third-generation phytases (~2,100–2,150 FYT/kg) [19]. This difference suggests greater enzymatic efficiency, allowing maximal biological responses to be achieved at lower inclusion levels.

All remaining carcass traits, including carcass yield, loin muscle depth, and lean meat content (both kg and %), positioned phytase-supplemented pigs as comparable to PC and superior to NC. Notably, for economically relevant traits such as HCW and lean meat percentage, all phytase inclusion levels resulted in responses equivalent to PC, indicating effective nutritional compensation [27,31]. The estimated optimal phytase inclusion level for maximizing lean meat percentage (~1,246 FYT/kg) further supports the dose–response nature of carcass traits under phytase supplementation [26,39].

In contrast, Dersjant-Li et al. [41] and Fandrejewski et al. [42] reported no significant effects of phytase on carcass characteristics when using lower inclusion levels and less severe mineral restriction. These discrepancies likely reflect differences in enzyme source, technological properties, dietary formulation, and experimental conditions [3,24,39]. Together, these findings reinforce that both enzyme characteristics and dietary context are critical determinants of the magnitude of phytase responses, including carcass traits. Moreover, previous studies using third-generation phytases often failed to detect improvements in carcass characteristics, even under moderate mineral restriction [27,31]. In contrast, the present results demonstrate clear improvements in carcass performance under more severe Ca and P restriction, indicating a greater biological potential of the evaluated phytase and supporting its superior efficiency relative to earlier-generation enzymes [4,12].

## Conclusions

The fourth-generation phytase evaluated in this study was effective in diets with reduced calcium and phosphorus levels for pigs across the nursery, growing, and finishing phases. Phytase supplementation restored growth performance and carcass traits to levels comparable to those observed in pigs fed diets adequately supplemented with these minerals, mitigating the negative effects of dietary mineral restriction. Across all evaluated phases, phytase inclusion consistently improved performance and carcass responses compared with the negative control, demonstrating its capacity to support productive efficiency under nutritionally challenging conditions. The optimal phytase inclusion range for both growth performance and carcass traits was consistently identified between 1,200 and 1,700 FYT/kg of feed. These results indicate that maximal biological responses can be achieved within this range under the conditions evaluated.
